# Simultaneous quantification of DNA damage and mitochondrial copy number by long-run DNA-damage quantification (LORD-Q)

**DOI:** 10.18632/oncotarget.20112

**Published:** 2017-08-10

**Authors:** Benjamin Dannenmann, Simon Lehle, Sebastian Lorscheid, Stephan M. Huber, Frank Essmann, Klaus Schulze-Osthoff

**Affiliations:** ^1^ Department of Molecular Medicine, Interfaculty Institute for Biochemistry, University of Tübingen, 72076 Tübingen, Germany; ^2^ Department of Radiation Oncology, University of Tübingen, 72076 Tübingen, Germany; ^3^ German Cancer Consortium (DKTK) and German Cancer Research Center (DKFZ), 69120 Heidelberg, Germany

**Keywords:** DNA damage, genotoxicity, mitochondrial DNA, LORD-Q, qPCR

## Abstract

DNA damage and changes in the mitochondrial DNA content have been implicated in ageing and cancer development. To prevent genomic instability and tumorigenesis, cells must maintain the integrity of their nuclear and mitochondrial DNA. Advances in the research of DNA damage protection and genomic stability, however, also depend on the availability of techniques that can reliably quantify alterations of mitochondrial DNA copy numbers and DNA lesions in an accurate high-throughput manner. Unfortunately, no such method has been established yet. Here, we describe the high-sensitivity long-run real-time PCR technique for DNA-damage quantification (LORD-Q) and its suitability to simultaneously measure DNA damage rates and mitochondrial DNA copy numbers in cultured cells and tissue samples. Using the LORD-Q multiplex assay, we exemplarily show that the mitochondrial DNA content does not directly affect DNA damage susceptibility, but influences the efficacy of certain anticancer drugs. Hence, LORD-Q provides a fast and precise method to assess DNA lesions, DNA repair and mtDNA replication as well as their role in a variety of pathological settings.

## INTRODUCTION

Human cells possess hundreds of mitochondria containing a circular genome of approximately 16 kilobase (kb) pairs. Although most mitochondria harbor several copies of mitochondrial DNA (mtDNA), replication can be highly variable and regulated in a cell type-specific manner. Moreover, mtDNA is highly susceptible to mutation, due to the continuous production of reactive oxygen species (ROS) during respiration, which cause oxidative lesions in the mtDNA [1]. Additionally, the DNA repair capacity for mtDNA is lower than that for nuclear DNA. Maintaining the integrity of mtDNA, however, is of utmost importance, since failures to properly repair mtDNA damage have been linked to ageing and a variety of human diseases [2]. Several studies have associated altered mtDNA copy numbers (mtDNAcn) with various types of cancer as well as with anti-cancer drug resistance [3–5]. Interestingly, the mtDNAcn has been proposed as a prognostic factor in lung cancer and also influences the risk of various other tumors, such as lymphomas or soft tissue sarcomas [6, 7]. Besides cancer, neurodegenerative diseases and depressive disorders have been associated with altered mtDNA content and DNA damage [8]. Furthermore, mtDNAcn alterations have been linked with impaired female fertility and childhood autism [9–11].

The growing interest in genotoxicity testing and research of mitochondrial dysfunctions in human disease has created an increasing demand for fast, robust and quantitative analytical methods for the assessment of DNA damage and mtDNA content. Unfortunately, most common methods are slow and cumbersome or have considerable limitations, as they are unsuitable for the sequence-specific detection of DNA lesions. Many methods to quantify DNA damage also depend on large amounts of sample DNA or labor-intensive normalization procedures, making these methods inapplicable for high-throughput analyses. Recently, we developed the long-run real-time PCR-based DNA damage quantification (LORD-Q) method [12]. LORD-Q is a PCR-based assay that relies on the principle that DNA lesions stall DNA polymerase, resulting in a decrease in the amount of PCR product. There are several advantages of using the LORD-Q assay. First, the assay is highly sensitive and sequence-specific, because it is primer-based and allows analysis of any gene *locus* in both the mitochondrial and nuclear genome. Moreover, LORD-Q needs very little sample DNA and enables the high-throughput quantification of DNA damage in long DNA templates of distinct gene *loci* of > 3 kb.

In the present study, we describe an optimized flexible LORD-Q procedure that can be used to simultaneously determine the number of DNA lesions and mtDNAcn. Using this adapted LORD-Q assay we investigated the relationship between mtDNAcn alterations and DNA damage in human cells following exposure to different genotoxic insults. Furthermore, we show that LORD-Q is capable to detect both DNA damage and mtDNAcn changes in tissue samples. Thereby, LORD-Q is not only suitable for genotoxicity testing and assessment of DNA repair processes, but also useful to assess pathological processes or drug actions that differentially affect copy number and lesions of mitochondrial or nuclear genomes.

## RESULTS AND DISCUSSION

### LORD-Q allows the simultaneous measurement of nuclear and mitochondrial DNA damage and mitochondrial DNA copy number

Due to an established role of mitochondrial dysfunction in aging, cancer and various other diseases as well as the recognized association between mtDNA damage and replication, it is important to elucidate the mechanisms underlying the maintenance of mtDNA integrity and mtDNA synthesis. Thus, development of methods to gene-specifically quantify DNA damage and the mtDNA copy number (mtDNAcn) might help to elucidate disease mechanisms and to provide targets for clinical interventions. So far, however, most conventional methods to detect DNA damage are of low sensitivity or detect DNA lesions in a global and sequence-independent manner.

We recently developed the LORD-Q assay, which allows the accurate quantification of DNA damage in distinct gene *loci* for the high-throughput assessment of DNA repair processes, genotoxicity testing and many other applications [12]. By using a novel rapid high-fidelity DNA polymerase, a second-generation fluorescent DNA dye, and by optimizing the PCR parameters, we were able to considerably increase the sensitivity of DNA damage detection and to establish a protocol for the quantification of DNA lesions in mitochondrial and nuclear probes of up to 4 kb length.

**Figure 1:**
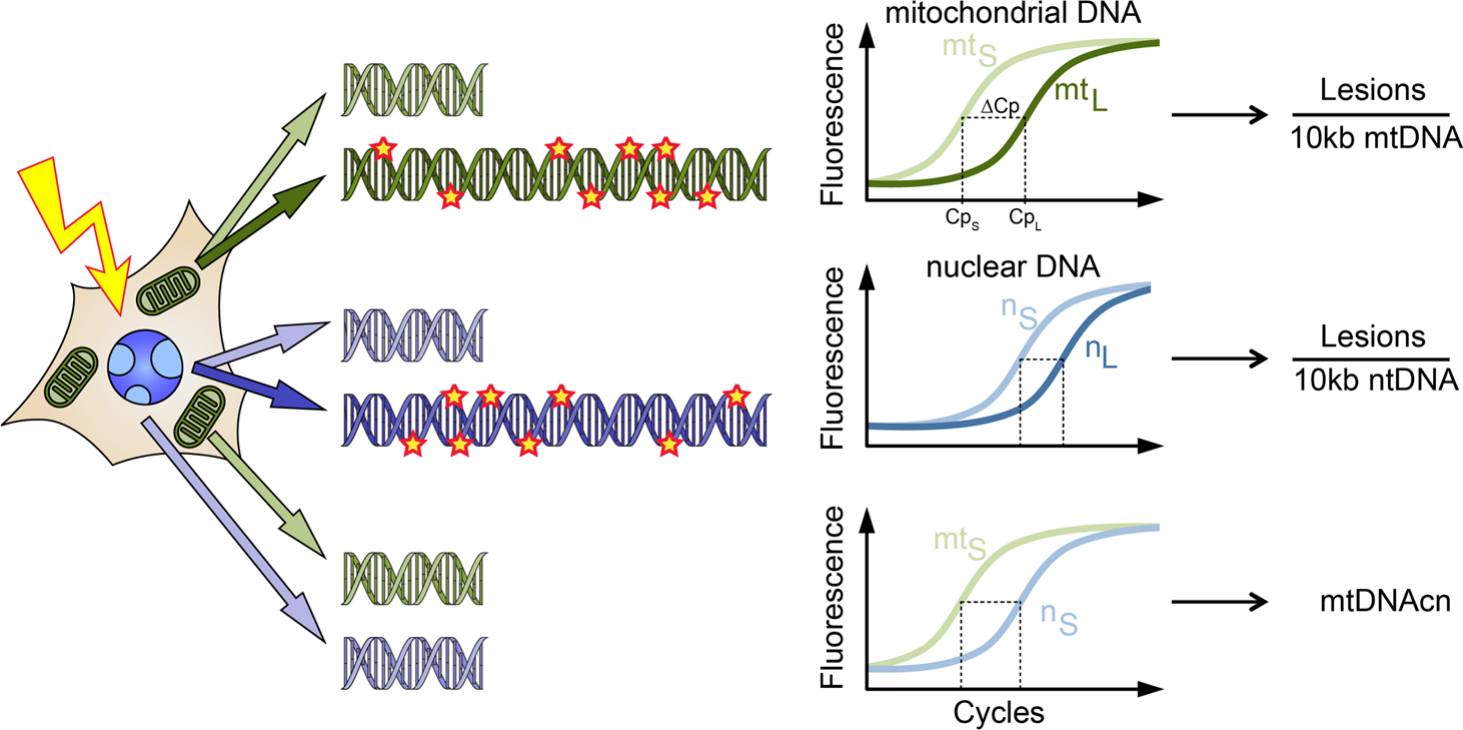
Schematic illustration of the LORD-Q assay allowing the simultaneous quantification of lesions in long DNA probes of mitochondrial (mt) and nuclear (n) genomes and the mtDNA copy number (mtDNAcn) Following treatment, e.g. with genotoxic agents, whole-cell DNA is isolated. The PCR amplification of long (L) template DNA sequences of 3–4 kb is inhibited by polymerase-stalling DNA lesions, resulting in a delayed exponential phase in the real-time PCR, which can be detected by the fluorescence signal of the DNA dye ResoLight. In contrast, short (S) probes of 40–70 bp, which serve as reference templates, are assumed to remain undamaged and are therefore amplified normally. Thereby, the average occurrence of DNA lesions can be calculated. Additionally, mtDNAcn can be calculated from the difference in C_p_ values of the short fragments of mitochondrial and nuclear DNA, taking the ploidy of the respective sample into account.

The present study describes an improved LORD-Q multiplex assay, which allows the simultaneous measurement of mtDNAcn together with the sequence-specific quantification of mitochondrial and nuclear DNA damage. The LORD-Q assay is based on the principle that DNA damage impedes DNA polymerase in the PCR reaction, resulting in decreased amounts of PCR product (Figure 1). For the assay, two fragments of different length are amplified in a real-time PCR reaction. A long DNA fragment of 3–4 kb from mitochondrial or nuclear DNA serves as experimental probe to detect DNA lesions. Since PCR amplification of this DNA template is inhibited by DNA lesions, the amount of the PCR amplification is inversely proportional to the amount of DNA damage. Assuming that DNA lesions are roughly distributed randomly and that each DNA base can be either damaged or undamaged, the formula used to calculate the DNA lesion rate can be derived from a Bernoulli equation. In order to calculate the probability of a single base being damaged (x), an undamaged reference is required. Because the probability of a DNA lesion to occur is proportional to the length of the fragment, a short fragment of ~ 50 to 70 bp, is assumed to be undamaged and serves as a normalization control.

$$\;\left(\underset{0}{Length}\right){\left(1-x\right)}^{Length}\;=\;{\left(1-x\right)}^{Length}$$

Subsequent data analysis is based on the measured crossing point (Cp) values for the long and the small fragment. Thus, the Cp values for amplification of the short (S) and the long fragment (L) of each sample, the amplification efficiencies (E) of the corresponding primer pairs and the amplicon length in bp of the long fragment allow the calculation of the average number of DNA lesions per 10 kb in the respective sample. An additional undamaged control sample serves as reference (Ref) for normalization of the results.

$$\frac{Lesions}{10\;kb}=\left[{\left(\frac{\frac{E_{L}{}^{Cp_{L}}}{E_{S}{}^{Cp_{S}}}}{{\left(\frac{E_{L}{}^{Cp_{L}}}{E_{S}{}^{Cp_{S}}}\right)}_{Ref}}\right)}^{\left(\frac{1}{length_{L}}\right)}-1\right]\times 10\;000$$

In the present study we extended the LORD-Q method to further investigate the link between DNA damage and mtDNAcn. If the LORD-Q assay is performed for a *locus* of both the mitochondrial and nuclear DNA, the shift between the exponential phases of the short fragment amplifications corresponds to the difference of mitochondrial and nuclear genome copies. Therefore, C_p_ values of the short reference fragments can be utilized to calculate mtDNAcn, provided that the ploidy p of the relevant gene in the sample is known.

$$mtDNAcn=p\times 2^{\left(Cp_{S(n)}-Cp_{S(mt)}\right)}$$

### Mitochondrial DNA copy number correlates with mitochondrial DNA damage

Oxidative stress is a continuous threat to the integrity of mtDNA. ROS trigger mtDNA damage that in a vicious circle can further enhance ROS generation [2, 13]. Moreover, elevated ROS levels induce replication of mtDNA [14], suggesting a protection-by-abundance mechanism that compensates for defects in mitochondria with damaged mtDNA. Therefore, we investigated a potential correlation of the copy number of mtDNA with DNA damage rates.

**Figure 2:**
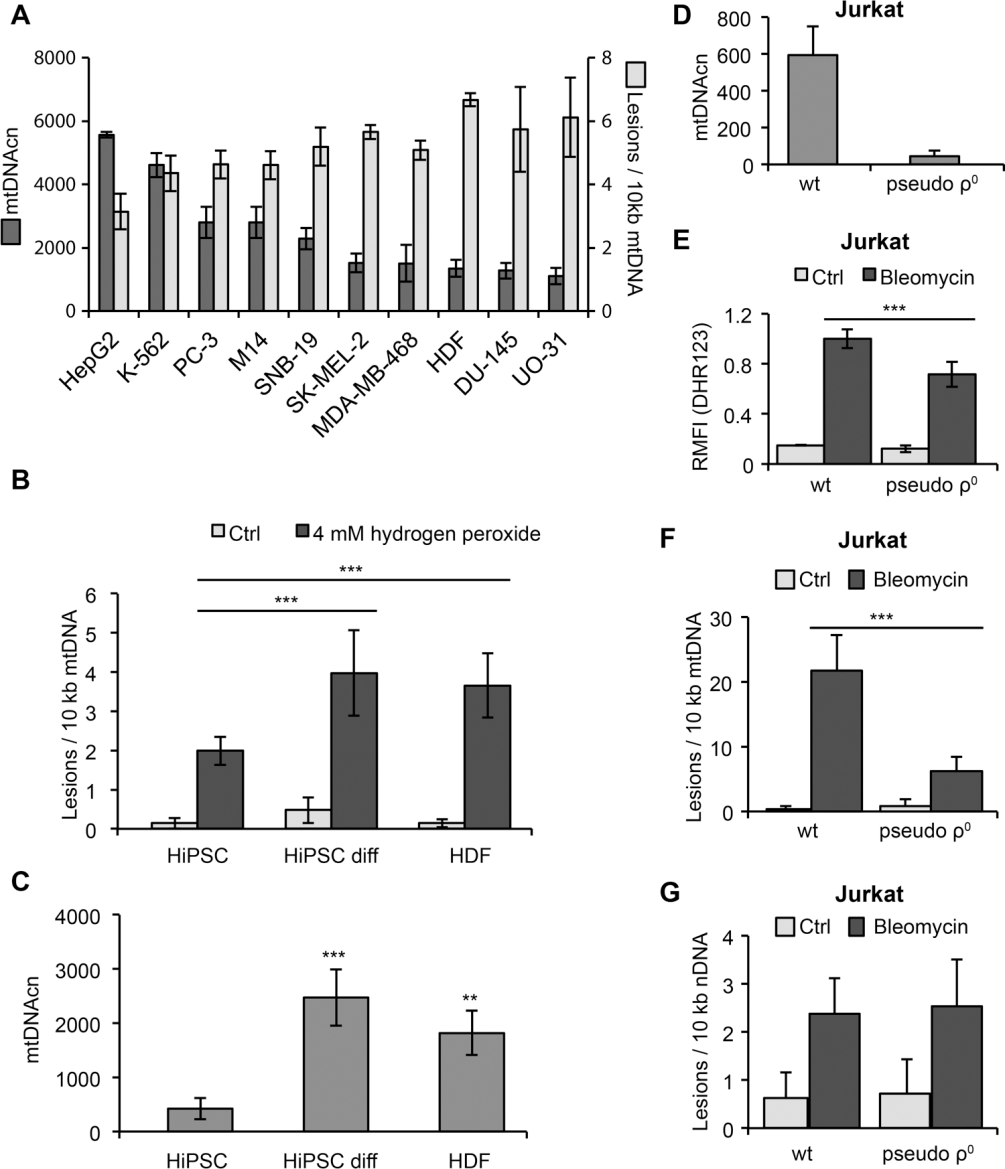
Correlation of mtDNA damage and copy number (**A**) Human dermal fibroblasts (HDF) and 9 different cancer cell lines from the NCI60 panel were irradiated with 10 mJ/cm^2^ UVC light and harvested immediately after irradiation. Damage of mtDNA and mtDNAcn were determined by LORD-Q analysis. (**B**) HiPSCs are less vulnerable to mtDNA damage than progenitor HDFs or differentiated HiPSCs after treatment with hydrogen peroxide for 5 min. (**C**) Reduced mtDNAcn of HiPSCs compared to HDFs and differentiated HiPSCs. (**D**) Depletion of mtDNA in Jurkat T cells cultured in the presence of 100 ng/mL ethidium bromide for 7 days. (**E**) Detection of ROS in wild-type (wt) and pseudo-ρ^0^ Jurkat cells following 1 hour of treatment with 100 μM bleomycin. The results were normalized to bleomycin-treated wild-type cells and indicate the relative median fluorescence intensity (RMFI) after staining with dihydrorhodamine 123 (DHR123). (**F**) Reduced mtDNA lesions in Jurkat pseudo-ρ^0^ cells compared to wild-type cells following 20 min of treatment with 100 μM bleomycin. (**G**) Lesion rates in nuclear DNA of Jurkat wild-type and pseudo-ρ^0^ cells following 20 min of treatment with 100 μM bleomycin do not significantly differ.

We first employed the LORD-Q method to analyze samples from human dermal fibroblasts (HDFs) and 9 cancer cell lines of the NCI60 panel representing different tumor entities. When cells were irradiated with UVC light to induce DNA damage, we generally detected less mtDNA damage in cells with higher mtDNAcn as compared to cells with less mtDNA copies (Figure 2A). For instance, HepG2 hepatoma cells, which contain almost 6000 mtDNA copies per cell, revealed the lowest number of mtDNA lesions, whereas U0-31 renal carcinoma cells with the lowest mtDNAcn were significantly more vulnerable to UVC-induced mtDNA damage. Hence, in this setting UVC-induced mtDNA damage appears to be inversely correlated with the number of mtDNA copies.

Several mechanisms are crucial for the maintenance of DNA integrity, including efficient DNA repair systems and high levels of antioxidants that inhibit genotoxic ROS formation [15–17]. To prevent harmful mutations, the maintenance of genomic stability must be very stringent. Pluripotent stem cells are particularly resistant to DNA damage due to their high levels of glutathione and strong expression of antioxidant enzymes, such as glutathione peroxidase-2 [18, 19]. We therefore compared the occurrence of mtDNA lesions in HDFs and human induced pluripotent stem cells (HiPSCs) that were either naïve or induced to undergo undirected differentiation by culture in the absence of FGF2. Indeed, a short-term exposure to hydrogen peroxide induced significantly less mtDNA damage in naïve HiPSCs compared to differentiated HiPSCs or HDFs (Figure 2B). In line with their reduced mitochondrial biogenesis [19], naïve HiPSCs contained lower mtDNAcn than differentiated HiPSCs or HDFs (Figure 2C). Thus, in contrast to the results described above in UV-irradiated cancer cells, in these cells the occurrence of oxidative stress-induced mtDNA damage appeared to correlate with mtDNAcn.

To further examine the relationship between mtDNAcn and DNA damage susceptibility, we depleted Jurkat cells of mtDNA (Figure 2D). Generation of mtDNA-depleted pseudo-ρ^0^ cells was accomplished either by incubation of cells with 2′,3′-dideoxycytidine (ddC), which is converted to the DNA polymerase γ inhibitor ddCTP, or by incubation with ethidium bromide, which intercalates into mtDNA and thereby inhibits mtDNA replication and transcription. Incubation of cells with either of these agents for 7 days resulted in a strong dose- and time-dependent depletion of mtDNA (Supplementary Figure 1). The depletion of mtDNA was associated with a drop of mitochondrial mass by 38%, as determined by flow cytometric staining with MitoTracker Green FM (Supplementary Figure 1). Importantly, when cells were treated with the chemotherapeutic drug bleomycin, Jurkat wild-type cells displayed increased ROS production and significantly more mtDNA damage compared to their mtDNA-depleted counterparts (Figure 2E, 2F). Intriguingly, the damage of nuclear DNA of pseudo-ρ^0^ cells was comparable to wild-type cells (Figure 2G). In line with previous studies [20], these results suggest that bleomycin preferentially induces mitochondrial rather than nuclear DNA lesions and that depletion of mtDNA can confer drug resistance. Moreover, bleomycin-induced ROS production appeared to correlate with the copy number and damage of the mtDNA. Whether mtDNAcn and mtDNA damage are causally linked to the formation and therapy resistance of tumors is still under debate and remains to be investigated in more detail in future studies. Certainly, LORD-Q will be a powerful method to provide further insight into these processes.

### Efficient analysis of tissue samples by LORD-Q

Traditional methods to determine DNA damage often require large quantities of sample DNA. The resulting need for large amounts of tissue limits the possibility to assess DNA damage in smaller organisms without pooling samples from several individuals. The requirement of large amounts of DNA also restricts analysis of clinical samples with limited availability. So far, the suitability of LORD-Q has only been tested in DNA samples derived from human cell culture [12]. As LORD-Q requires only nanogram quantities of isolated DNA, we tested its applicability for the simultaneous analysis of mtDNA damage and copy number in murine tissue samples. For the animal experiments we chose γ-irradiation as a genotoxic stimulus, as it was reported to induce DNA damage and changes in mtDNAcn [21, 22].

We γ-irradiated mice with 3 Gy or 6 Gy and isolated samples of whole-body irradiated and control mice from several organs that differ in their radiation sensitivity, i.e. brain, spleen, liver and bone marrow. To account for different DNA repair capacities, the tissue samples were isolated from the mice 3 and 24 hours post-irradiation. After excision of the organs and isolation of bone marrow, we analyzed DNA damage in mitochondrial and nuclear DNA and determined mtDNAcn using the LORD-Q assay.

**Figure 3:**
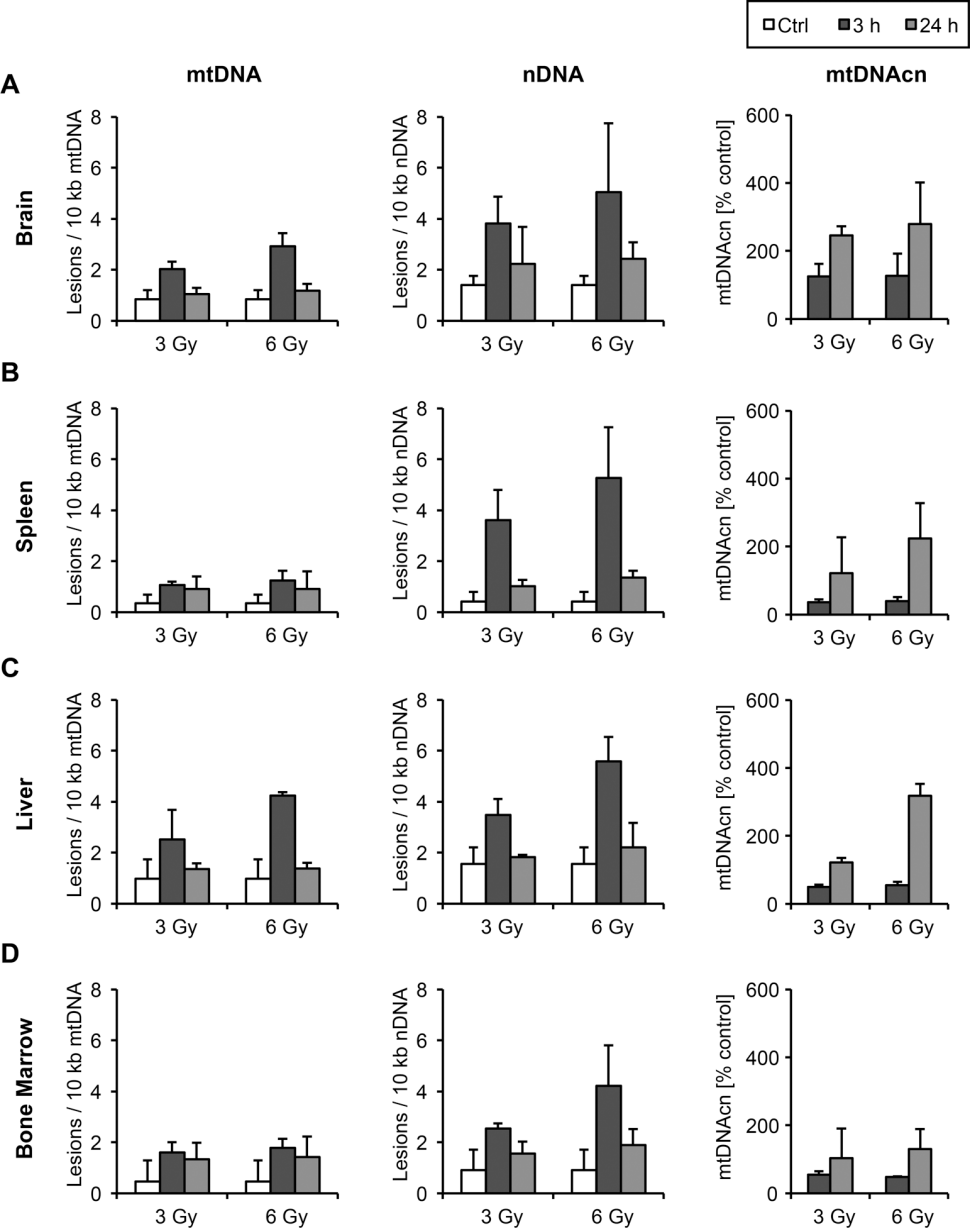
Mitochondrial and nuclear DNA damage and mtDNAcn in mice following ionizing irradiation Mice were exposed to 3 Gy and 6 Gy of ionizing irradiation, before mitochondrial and nuclear DNA lesions as well as alterations of mtDNAcn were measured by LORD-Q in isolated brain (**A**), spleen (**B**), liver (**C**) and bone marrow (**D**) at 3 and 24 hours post-irradiation (*n* ≥ 3). The mtDNAcn is given as the percentage relative to the respective tissue of untreated control mice.

Different organs of the irradiated mice exhibited different levels of DNA damage compared to samples from non-irradiated animals. In brain tissue, mtDNA showed a dose-dependent increase of 2–3 lesions per 10 kb 3 hours post-irradiation, which were mostly repaired within 24 hours (Figure 3A). Nuclear DNA of brain tissue displayed higher lesion rates (4 – 5 per 10 kb) as compared to mtDNA. The mtDNAcn in irradiated brain cells showed no significant change within the first 3 hours post-treatment, however, 24 hours after irradiation mtDNAcn was increased more than 2-fold as compared to untreated control cells (Figure 3A).

Compared to brain, we detected very low mtDNA lesion rates in spleen, whereas nuclear DNA damage was comparable in both tissues (Figure 3B). Nuclear DNA lesions in spleen, however, seemed to be more efficiently repaired compared to brain samples. The copy numbers of mtDNA showed an initial decrease 3 hours post-irradiation and increased in a dose-dependent manner during 24 hours post-irradiation (Figure 3B). Higher doses of ionizing radiation and DNA damage clearly resulted in enhanced replication of mtDNA.

Liver tissue showed comparably high lesion rates in mtDNA 3 hours post-treatment, which were efficiently repaired within 24 hours (Figure 3C). Nuclear DNA damage levels were slightly higher than in mtDNA, but comparable to nuclear DNA damage in the brain. The copy numbers of mtDNA initially decreased, but became elevated up to 3-fold after 24 hours (Figure 3C). Bone marrow accumulated relatively few mtDNA lesions, and also nuclear DNA appeared less vulnerable compared to other tissues (Figure 3D). Following an initial decay, mtDNAcn in bone marrow were restored within 24 hours. Thus, LORD-Q demonstrates a dose-dependent increase of lesions in both the mitochondrial and nuclear DNA. After 24 hours DNA lesion rates in all samples were only marginally higher compared to the untreated controls, reflecting an efficient DNA repair.

Altogether, this study demonstrates a high suitability of LORD-Q for the simultaneous quantification of DNA damage and mtDNAcn in cultured cells as well as in tissue samples. Both DNA damage and mtDNAcn alterations have been associated with cancer formation, altered tumor metabolism and resistance to anticancer drugs. Hence, LORD-Q provides a valuable tool for the evaluation of new chemotherapeutic drugs targeting these parameters. Abnormalities of mtDNAcn have been also proposed as a prognostic marker in certain tumors, which could be conveniently analyzed in more detail using the LORD-Q method. In addition, the fields of ageing research and reproduction medicine might benefit from a sensitive assay capable of simultaneously measuring DNA damage and mtDNA ploidy. Finally, LORD-Q might be a valuable tool for large-scale genotoxicity screening programs, for instance for the European Union REACH regulation, which addresses the impact of chemicals on both human health and the environment. Thus, LORD-Q is a unique technique that enables the sequence-specific quantification of DNA damage and mtDNA copies and thereby allows numerous applications in oncology, toxicology and pharmaceutical research.

## MATERIALS AND METHODS

### Animal experimentation

Male C57/BL6 mice were used at 6 to 11 weeks of age. Ionizing irradiation was performed using a Philips SL25 linear accelerator (Philips; Amsterdam, The Netherlands) with irradiation rates of 1.3 Gy per minute. Mouse work was performed in accordance with the German law guidelines of animal care, as permitted by regional authorities (Regierungspräsidium Tübingen, no. IB 1/16). Organs were excised at the indicated times post-irradiation and bone marrow was isolated as described [23].

### Cell culture and genotoxic treatment

Human Jurkat T lymphocytes were depleted of mtDNA by cultivation of cells in the presence of 2′-3′-dideoxycytidine (ddC) or ethidium bromide [24, 25]. The selection medium was further supplemented with 1 mM sodium pyruvate and 100 μg/mL uridine. Human induced pluripotent stem cells (HiPSCs) were generated by transduction of human dermal fibroblasts (HDFs) with the Yamanaka retroviral cocktail for the expression of OCT4, SOX2, KLF4 and c-MYC reprogramming factors. Cells were cultured and validated for stem cell properties as described [12]. Undirected differentiation of HiPSCs was performed by culture in the presence of FCS and absence of FGF2 for 30 days. Cells were treated with hydrogen peroxide and bleomycin or UV-irradiated as described [18].

### DNA isolation

DNA was purified from cultured cells and tissue using the DNeasy Blood & Tissue kit (Qiagen, Hilden, Germany). Tissue lysis in ATL buffer (Qiagen) was carried out for 30 min. DNA concentration was determined spectrometrically using a Nanodrop 1000 photometer (Peqlab, Erlangen, Germany). DNA was diluted to 5 ng/μL with buffer AE prior to use.

### Flow cytometry

To measure intracellular ROS levels, cells were stained with 1 μM dihydrorhodamine 123 (Sigma-Aldrich; St. Louis, MO) and analyzed by flow cytometry [18, 26]. Relative cellular content of mitochondria was determined using MitoTracker Green FM (Thermo Fisher, Waltham, MA) essentially as described [27].

### LORD-Q assay

Measurement of mitochondrial and nuclear DNA damage was performed by the LORD-Q method as originally described [12]. For each analyzed genomic or mitochondrial gene *locus* a long DNA fragment (~3000 – 4000 bp) was used as sensor for DNA lesions and an internally nested short fragment (~50 – 70 bp) that was considered as undamaged served as reference. The nested intact reference was efficiently PCR-amplified, whereas amplification of the large sensor is inhibited by DNA lesions including abasic sites, thymine dimers, strand breaks and oxidative lesions [12]. Upon induction of DNA damage, the exponential amplification phase for the damage-sensitive large fragment is reached later as compared to the nested intact reference. The difference in crossing point values (C_p_) therefore allows calculation of the average incidence of lesions per bp.

Initially, the replication efficiency of each primer pair was determined using a standard dilution of whole-cell DNA as described [12]. Then, real-time PCR was carried out in 96-well or 384-well plates with each well containing a reaction volume of 20 μL and 10 μL, respectively. The reaction mixture contained 2.5 ng/μL isolated sample DNA, 1 x KAPA2G HS Polymerase ReadyMix (Peqlab), 0.0016 x ResoLight dye (Roche, Basel, Switzerland) and 500 nM of HPLC-purified forward and reverse primers (Sigma-Aldrich), respectively.

Cycling conditions were as follows: a pre-incubation phase of 5 min at 95°C was followed by up to 60 cycles of 10 s at 95°C, 10 s at 60°C and 1 s at 72°C for small amplicons or 135 s for large amplicons, respectively. The reactions were carried out in a LightCycler 480 II system (Roche). Samples were measured in triplicates (96-well plates) or quadruplicates (384-well plates). C_p_ values were calculated using the LightCycler 480 software and replicates were averaged. All primers were designed to match the requirements of LORD-Q and are listed in Supplementary Table 1. For detection of nuclear DNA damage promoter sequences of the human and murine *COL1A1 locus* were analyzed. Data is depicted as mean ± standard deviation from at least three independent experiments.

## SUPPLEMENTARY MATERIALS FIGURE AND TABLE



## References

[1] Sawyer DE, Van Houten B (1999). Repair of DNA damage in mitochondria. Mutat Res.

[2] Balaban RS, Nemoto S, Mitochondria Finkel T (2005). Oxidants, and Aging. Cell.

[3] Reznik E, Miller ML, Şenbabaoğlu Y, Riaz N, Sarungbam J, Tickoo SK, Al-Ahmadie HA, Lee W, Seshan VE, Hakimi AA, Sander C (2016). Mitochondrial DNA copy number variation across human cancers. eLife.

[4] van Gisbergen MW, Voets AM, Starmans MHW, de Coo IFM, Yadak R, Hoffmann RF, Boutros PC, Smeets HJM, Dubois L, Lambin P (2015). How do changes in the mtDNA and mitochondrial dysfunction influence cancer and cancer therapy? Challenges, opportunities and models. Mutat Res Rev Mutat Res.

[5] Mei H, Sun S, Bai Y, Chen Y, Chai R, Li H (2015). Reduced mtDNA copy number increases the sensitivity of tumor cells to chemotherapeutic drugs. Cell Death Dis.

[6] Xu H, He W, Jiang HG, Zhao H, Peng XH, Wei YH, Wei JN, Xie CH, Liang C, Zhong YH, Zhang G, Deng D, Zhou YF (2013). Prognostic value of mitochondrial DNA content and G10398A polymorphism in non-small cell lung cancer. Oncol Rep.

[7] Mi J, Tian G, Liu S, Li X, Ni T, Zhang L, Wang B (2015). The Relationship Between Altered Mitochondrial DNA Copy Number And Cancer Risk: A Meta-Analysis. Sci Rep.

[8] Chang CC, Jou SH, Lin TT, Lai TJ, Liu CS (2015). Mitochondria DNA Change and Oxidative Damage in Clinically Stable Patients with Major Depressive Disorder. PLoS One.

[9] Hendriks WK, Colleoni S, Galli C, Paris DB, Colenbrander B, Roelen BA, Stout TA (2015). Maternal age and *in vitro* culture affect mitochondrial number and function in equine oocytes and embryos. Reprod Fertil Dev.

[10] Otten ABC, Smeets HJM (2015). Evolutionary defined role of the mitochondrial DNA in fertility, disease and ageing. Hum Reprod Update.

[11] Chen S, Li Z, He Y, Zhang F, Li H, Liao Y, Wei Z, Wan G, Xiang X, Hu M, Xia K, Chen X, Tang J (2015). Elevated mitochondrial DNA copy number in peripheral blood cells is associated with childhood autism. BMC Psychiatry.

[12] Lehle S, Hildebrand DG, Merz B, Malak PN, Becker MS, Schmezer P, Essmann F, Schulze-Osthoff K, Rothfuss O (2014). LORD-Q: a long-run real-time PCR-based DNA-damage quantification method for nuclear and mitochondrial genome analysis. Nucleic Acids Res.

[13] Indo HP, Davidson M, Yen HC, Suenaga S, Tomita K, Nishii T, Higuchi M, Koga Y, Ozawa T, Majima HJ (2007). Evidence of ROS generation by mitochondria in cells with impaired electron transport chain and mitochondrial DNA damage. Mitochondrion.

[14] Lee HC, Yin PH, Lu CY, Chi CW, Wei YH (2000). Increase of mitochondria and mitochondrial DNA in response to oxidative stress in human cells. Biochem J.

[15] Balendiran GK, Dabur R, Fraser D (2004). The role of glutathione in cancer. Cell Biochem Funct.

[16] Dröge W, Schulze-Osthoff K, Mihm S, Galter D, Schenk H, Eck HP, Roth S, Gmünder H (1994). Functions of glutathione and glutathione disulfide in immunology and immunopathology. FASEB J.

[17] Schulze-Osthoff K, Bauer MK, Vogt M, Wesselborg S (1997). Oxidative stress and signal transduction. Int J Vitam Nutr Res.

[18] Dannenmann B, Lehle S, Hildebrand DG, Kübler A, Grondona P, Schmid V, Holzer K, Fröschl M, Essmann F, Rothfuss O, Schulze-Osthoff K (2015). High glutathione and glutathione peroxidase-2 levels mediate cell-type-specific DNA damage protection in human induced pluripotent stem cells. Stem Cell Rep.

[19] Dannenmann B, Lehle S, Essmann F, Schulze-Osthoff K (2015). Genome surveillance in pluripotent stem cells: Low apoptosis threshold and efficient antioxidant defense. Mol Cell Oncol.

[20] Brar SS, Meyer JN, Bortner CD, Van Houten B, Martin WJ (2012). Mitochondrial DNA-depleted A549 cells are resistant to bleomycin. AJP Lung Cell Mol Physiol.

[21] Zhang H, Maguire D, Swarts S, Sun W, Yang S, Wang W, Liu C, Zhang M, Zhang D, Zhang L, Zhang K, Keng P, Zhang L (2009). Replication of murine mitochondrial DNA following irradiation. Adv Exp Med Biol.

[22] Malakhova L, Bezlepkin VG, Antipova V, Ushakova T, Fomenko L, Sirota N, Gaziev AI (2005). The increase in mitochondrial DNA copy number in the tissues of gamma-irradiated mice. Cell Mol Biol Lett.

[23] Hörber S, Hildebrand DG, Lieb WS, Lorscheid S, Hailfinger S, Schulze-Osthoff K, Essmann F (2016). The Atypical Inhibitor of NF-κB, IκBζ, Controls Macrophage Interleukin-10 Expression. J Biol Chem.

[24] King MP, Attardi G (1989). Human cells lacking mtDNA: repopulation with exogenous mitochondria by complementation. Science.

[25] Schulze-Osthoff K, Beyaert R, Vandevoorde V, Haegeman G, Fiers W (1993). Depletion of the mitochondrial electron transport abrogates the cytotoxic and gene-inductive effects of TNF. EMBO J.

[26] Hassan M, Alaoui A, Feyen O, Mirmohammadsadegh A, Essmann F, Tannapfel A, Gulbins E, Schulze-Osthoff K, Hengge UR (2008). The BH3-only member Noxa causes apoptosis in melanoma cells by multiple pathways. Oncogene.

[27] Essmann F, Pohlmann S, Gillissen B, Daniel PT, Schulze-Osthoff K, Jänicke RU (2005). Irradiation-induced translocation of p53 to mitochondria in the absence of apoptosis. J Biol Chem.

