# Supplementary material for: Simultaneous quantification of DNA damage and mitochondrial copy number by long-run DNA-damage quantification (LORD-Q)
Source: Oncotarget. 2017 Aug 10;8(68):112417–25. doi: 10.18632/oncotarget.20112 (PMC5762520; doi:10.18632/oncotarget.20112)
Supplement: Supplementary file 1 [file oncotarget-08-112417-s001.pdf]

Simultaneous quantification of DNA damage and mitochondrial copy number by long-run DNA-damage quantification (LORD-Q)

SUPPLEMENTARY MATERIALS

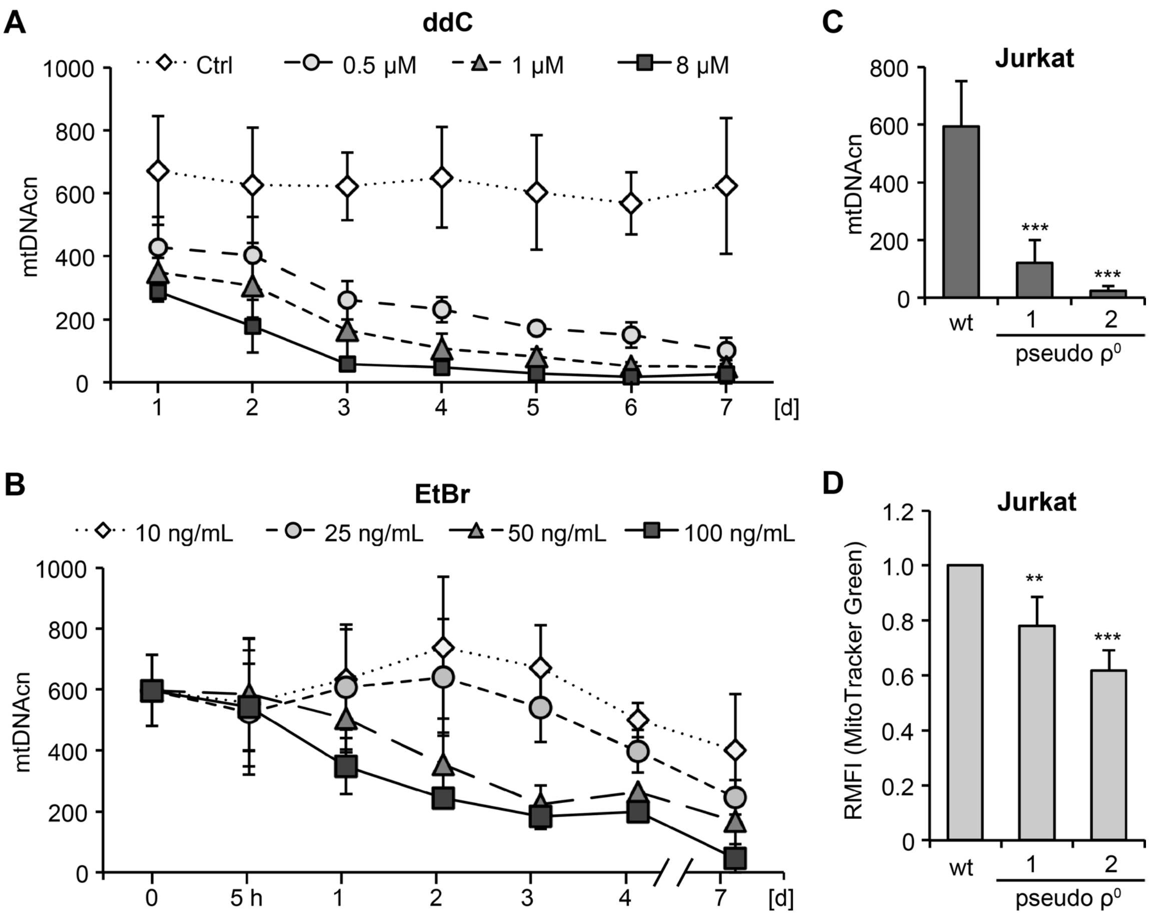

Supplementary Figure 1: Depletion of mtDNA in Jurkat T cells. Cells were cultured in the presence of the indicated concentrations of (**A**) dideoxycytidine (ddC) or (**B**) ethidium bromide (EtBr), resulting in an up to 90% reduction of mtDNAcn within the first 3 days. (**C**) mtDNAcn of two pseudo-ρ^0 Jurkat cell lines that were independently generated by incubation with dideoxycytidine or ethidium bromide. (**D**) The Jurkat pseudo-ρ^0 cell clones were incubated with the mitochondria-specific dye MitoTracker Green FM and analyzed by flow cytometry. The reduction of fluorescence intensity indicating a loss of mitochondrial mass correlated with a drop of the mtDNAcn.

Supplementary Table 1: LORD-Q primers applied in DNA damage quantification experiments

Locus	Base pairs	Primer Denotation	Primer Sequence
mtDNA (L) human	3724	H.mtDNA.L.F	5′-ATCGTAGCCTTCTCCACTTC-3′
		H.mtDNA.R	5′-TGGTTAGGCTGGTGTTAGGG-3′
mtDNA (S) human	50	H.mtDNA.S.F	5′-GGCCACAGCACTTAAACACA-3′
		H.mtDNA.R	5′-TGGTTAGGCTGGTGTTAGGG-3′
mtDNA (L) mouse	3921	MM.mtDNA.F	5′-TCCTACTGGTCCGATTCCAC-3′
		MM.mtDNA.L.R	5′-CGGTCTATGGAGGTTTGCAT-3′
mtDNA (S) mouse	74	MM.mtDNA.F	5′-TCCTACTGGTCCGATTCCAC-3′
		MM.mtDNA.S.R	5′-GGCTCCGAGGCAAAGTATAG-3′
COL1A1 (L) human	3578	H.COL1A1.L.F	5′-ATTATCGGGACATCGGTGAA-3′
		H.COL1A1.R	5′-CCACCAAAGCTTTCTTCTGC-3′
COL1A1 (S) human	52	H.COL1A1.S.F	5′-TGCAGGGTGAGAAACATGAC-3′
		H.COL1A1.R	5′-CCACCAAAGCTTTCTTCTGC-3′
Col1a1 (L) murine	2637	MM.col1a1.L1.F	5′-CCGTTTGTCCCATTACTGCT-3′
		MM.col1a1.L1.R	5′-AGCAAGGACGAGGACTTTGA-3′
Col1a1 (S) murine	60	MM.col1a1.S.F	5′-AAAGTGGGAATCTGGACACG-3′
		MM.col1a1.S.R	5′-CAGAGGCCTTATTTCATTTTCG-3′
